# Chemical Modeling of Acid-Base Properties of Soluble Biopolymers Derived from Municipal Waste Treatment Materials

**DOI:** 10.3390/ijms16023405

**Published:** 2015-02-04

**Authors:** Silvia Tabasso, Silvia Berto, Roberta Rosato, Janeth Alicia Tafur Marinos, Marco Ginepro, Vincenzo Zelano, Pier Giuseppe Daniele, Enzo Montoneri

**Affiliations:** 1 Department of Chemistry, University of Turin, Via Pietro Giuria 7, I-10125 Torino, Italy; E-Mails: silvia.tabasso@unito.it (S.T.); rosato.roberta1@gmail.com (R.R.); janethalicia.tafurmarinos@unito.it (J.A.T.M.); marco.ginepro@unito.it (M.G.); vincenzo.zelano@unito.it (V.Z.); piergiuseppe.daniele@unito.it (P.G.D.); 2 Biowaste Processing, Via XXIV Maggio 25, 37126 Verona, Italy; E-Mail: enzo.montoneri@gmail.com

**Keywords:** biopolymers, biowastes, chemical model, potentiometry, protonation constants, NMR

## Abstract

This work reports a study of the proton-binding capacity of biopolymers obtained from different materials supplied by a municipal biowaste treatment plant located in Northern Italy. One material was the anaerobic fermentation digestate of the urban wastes organic humid fraction. The others were the compost of home and public gardening residues and the compost of the mix of the above residues, digestate and sewage sludge. These materials were hydrolyzed under alkaline conditions to yield the biopolymers by saponification. The biopolymers were characterized by ^13^C NMR spectroscopy, elemental analysis and potentiometric titration. The titration data were elaborated to attain chemical models for interpretation of the proton-binding capacity of the biopolymers obtaining the acidic sites concentrations and their protonation constants. The results obtained with the models and by NMR spectroscopy were elaborated together in order to better characterize the nature of the macromolecules. The chemical nature of the biopolymers was found dependent upon the nature of the sourcing materials.

## 1. Introduction

Municipal biowastes (MBW) are a readily available cost-effective source [1] of soluble biopolymers (henceforth SBOs, Soluble Bio-Organic substances). These products are composed by a mixture of molecules with 67–463 kg·mol^−1^ molecular weight and 6–53 polydispersity index. They contain long aliphatic C chains and aromatic rings substituted by a wide variety of acid and basic functional groups of different strength. These organic moieties are the likely memory of the main constituents of the sourcing bio-organic refuse matter, which are not completely mineralized during aging under fermentation conditions. As a result of the biological origin of their sourcing materials, in addition to the high polydispersity index, the entire molecular pool is expected to be heterogeneous also for chemical composition. By these features SBO bear chemical similarities with soil and water humic substances (HS) formed under longer aging.

The chemical compositions and properties of SBOs may be different owing to the MBW sourcing material. Recently, several papers have reported that SBO from different sources can find application in diversified fields; e.g., in the formulation of detergents textile dyeing baths, flocculants, dispersants and binding agents for ceramics manufacture [1], emulsifiers [2], auxiliaries for soil/water remediation [3,4,5] and enhanced oil recovery [6], nanostructured materials for chemical [7,8] and biochemical catalysis [9], plastic materials [10], soil fertilizers and plant biostimulants for agriculture [11] and animal feed supplements [12,13]. The wide range of applications arises from the variety of C moieties distributed over the macromolecular pool constituting the SBOs. The C moieties are the memories of the protein, fats, and polysaccharide and lignin proximates constituting the biowastes from which the SBO are obtained. These chemical features are associated to the SBO properties as surfactants, agents for sequestering or carrying small molecules and mineral ions in solution, photosensitizers and reactive biopolymers. Further understanding of the chemical nature of SBOs might help product development for the above uses and other new specific applications. In this work we report the study of the acid-base behavior of these substances. This specific molecular feature has not been studied so far. Yet, in some of the above applications, the performance of SBO has been found to connect to their acidic sites. In agriculture [11], for instance, the SBOs have been found capable to bind and transport soluble mineral nutrients through the soil to enhance plant growth. In the photoremediation of industrial wastewaters [4,5], the capacity of SBOs to bind and keep Fe ions in solution under non-acidic conditions has been claimed enhancing the mineralization of organic pollutants upon exposure to solar light. Specific studies of the acid-base behavior of SBOs are likely to disclose potential for new applications. For instance, due to their protogenic sites, the SBOs may perform as bioadsorbents for the removal of metal ions by exchanging protons with metal ions. The exploitation of the capacity of these substances to bind metal ions at neutral and alkaline pH, and of the property to precipitate at acid pH, may prospect an interesting process for the remediation of water streams containing toxic metals. Other biopolymers of natural origin, such as soil and water humic substances (HS), have been studied for their capacity to exchange protons with metal ions [14,15,16,17,18,19,20,21]. These substances bear chemical similarities with the above SBOs. However, for large scale uses in environmental remediation practices, the SBOs are potentially favored for the cost effectiveness and the environmental benefits arising from the exploitation of their sourcing materials [1].

The evaluation of the proton exchange capacity of bio-macromolecules was not easy. The modeling of protonation equilibria is very complicated, particularly in the case of natural polyelectrolytes with heterogeneous chemical compositions and structures. The literature comprises many articles in which different approaches are proposed to describe the acid-base properties of HS [14,15,16,17,18,19,20,21,22]. The main difficulties when dealing with the protonation parameters of this type of polyelectrolytes are the pH drift during titration and the hysteresis between tandem forward and reverse titration curves. The hysteresis may be caused by base-catalyzed hydrolyses of esters and amides. It can also be due to the dependence of acidity constants on molecular conformation and charge [19,21,22]. The conformation of the macromolecules changes during the titration for the electrostatic repulsion of negative charges, and this affects the proton exchange capacity of functional groups. Moreover, the protonation parameters depend on the effective charge of the polyanion, which is affected by the dissociation degree of the macromolecule [21,22]. Thus, different methods have been proposed and tested for the evaluation of protonation parameters connected to carboxylic and phenolic functional groups in HS.

In this work, alkalimetric and acidimetric titrations of SBO solutions were conducted and were elaborated in order to propose a chemical model that can describe the proton-binding capacity of the macromolecules. The application of software that employs an iterative and convergent numerical method, which is based upon the linear combination of the mass balance equations, allows calculating the acidic sites concentrations and their protonation constants. A discrete model and a diprotic-like model were applied. The first one supposes that a macromolecule contains a series of discrete sites with different dissociation constant values [15]. The diprotic-like model, proposed by Crea* et al.* [22] assumes that the binding capacity of the carboxylic acid groups is described by two protonation constants, whereas the phenolic group behaves as a second ligand in the alkaline pH range. Both model types assume that the protonation parameters are independent of the dissociation degree. Hereinafter, the concentration values of carboxylic groups obtained with the modeling approach are discussed in relation to previously reported values [23] obtained by NMR and elemental analyses.

## 2. Results and Discussion

### 2.1. Chemical Composition and Solution Behavior of Soluble Bio-Organic Substances (SBOs)

In this study three different SBOs, called FORSUD, CVDF, and CV, were investigated. These products were obtained from three different materials. As reported in the Section 3.2, the CV and CVDF were sourced from composts, whereas FORSUD was isolated from the anaerobic fermentation digestate. According to the chemical process described in the same Section 3.2, the SBOs are hydrolysates with molecular cut-off above 5000 Da. They contain water-soluble polymeric molecules, which may range up [23] to several hundred kg·mol^−1^. Due to their complexity, SBO cannot be characterized as well as synthetic molecules. Their chemical nature can at best be identified by the concentration of C types and functional groups reported in Table 1. The behavior of these polymeric substances cannot be understood as well as synthetic single molecules. The C types and functional groups reported in Table 1 are likely to be not homogeneously distributed over the entire molecular pool. The data indicate that the SBOs contain three main groups capable to bind protons or metal ions;* i.e.*, carboxyl, phenol and amino groups. Indeed, the reduction of the mineral content of these products required a first treatment with HCl only, followed by a second treatment with HF.

For the purpose of this work, it was necessary to understand first the solution behavior of the SBO. From the presence of both lipophilic and hydrophilic, the SBOs were expected to perform typically as surfactants. The behavior of simple surfactants’ molecules, such as sodium dodecylbenzene sulfonate (SDBS), is well known [23]. At low concentration, SDBS molecules occupy the air-water interphase, with their polar hydrophilic heads pointing toward the water phase and their lipophilic tails directed toward the air phase. At high concentration, they aggregate to form micelles in the bulk water phase. These may have different shapes, but the polar head are exposed to the water phase and the lipophilic tails are oriented toward the micelle core. Conformation details for the complex mixtures of SBO macromolecules cannot be envisioned as straightforwardly. Two virtual fragments for these polymeric molecules, containing the C types and functional groups listed in Table 1, are reported in a previous work [23]. Starting from these fragments, one could imagine that at low concentrations the single macromolecules occupy the air-water interphase and acquire a more or less flat configuration to point their hydrophilic heads toward the water phase. At higher concentration, upon saturating the air-water interphase, they would occupy the bulk water phase. However, due to their polymeric nature, two conformations are possible in the bulk water phase [24]. The single molecules could be arranged in coil form to yield pseudo-micelles with the external hydrophilic surface pointing toward the water phase and the inner lipophilic molecular segments away from it. At higher concentrations, the single pseudo-micelles might interact to yield micellar aggregates. The aggregation of molecules in pseudo-micelles conformation, due to increasing concentration above 2 g·L^−1^, would occur through H-bonding interaction between the external hydrophilic groups of the pseudo-micelles. As a consequence, in the pseudo-micelles’ aggregates some of the hydrophilic phenol and carboxylic groups might end up inside the aggregate core and become less accessible by the titrant. This situation has been proposed based on the results of dynamic light scattering measurements reported in previous work [24]. The size of SBO macromolecules has been found to be more sensitive to pH at 0.2 g·L^−1^ than at 2 g·L^−1^ concentration. At 0.2 g·L^−1^ SBO concentration, the molecular hydrodynamic diameter (*D*_h_) has been found to change from 71–73 nm at pH 5 to 128–168 nm at pH 7 and to 137–218 nm at pH 13, whereas for the 2 g·L^−1^ sample *D*_h_ changes from 90–97 nm at pH 5 to 101–123 nm at pH 7–13. The increase of *D*_h_ is likely due to the increasing ionization and repulsion between carboxylate groups occurring upon increasing pH. By comparison at the same pH, the ionization and the repulsion in the inner core of the micellar aggregates, formed at concentration above 2 g·L^−1^, is less due to the lower accessibility of the alkaline cation into the micellar core. This explains the fact that at 0.2 g·L^−1^, compared to 2 g·L^−1^ concentration, the *D*_h_ sensitivity* versus* temperature was found up to twice as high, whereas the sensitivity* versus* pH was four to eight times higher. Under these circumstances, it was deemed that a more reliable assessment of the number and nature of SBO protogenic sites could be obtained from measurements performed below the SBO critical micellar concentration (Table 1).

### 2.2. Proton Binding Capacity of SBOs

**Figure 1:**
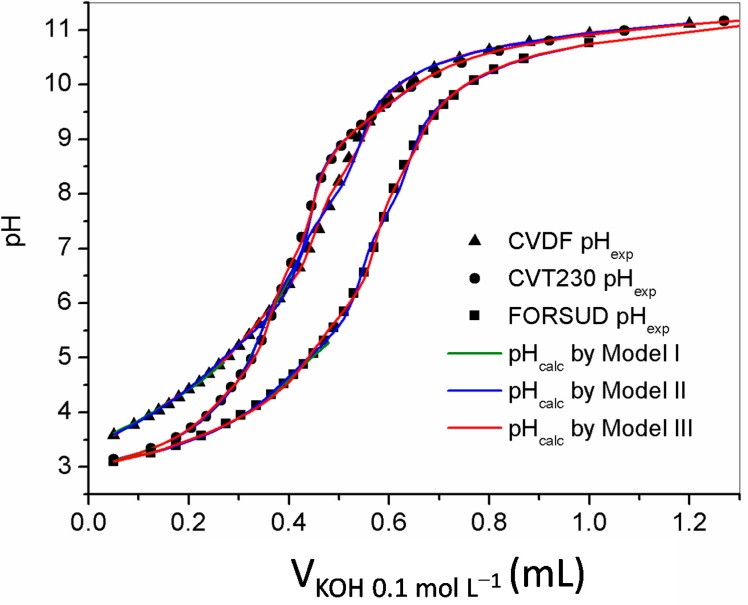
Examples of experimental titration points (scatter plots) and calculated titration curves (colored curves) for CVDF, CVT230 and FORSUD, under the following conditions: 25 mL of solution containing 0.5 g·L^−1^ SBO and 0.1 mol·L^−1^ TEACl, titrated with 0.1 mol·L^−1^ KOH. In the graph, one titration point was reported every other two.

**Figure 2:**
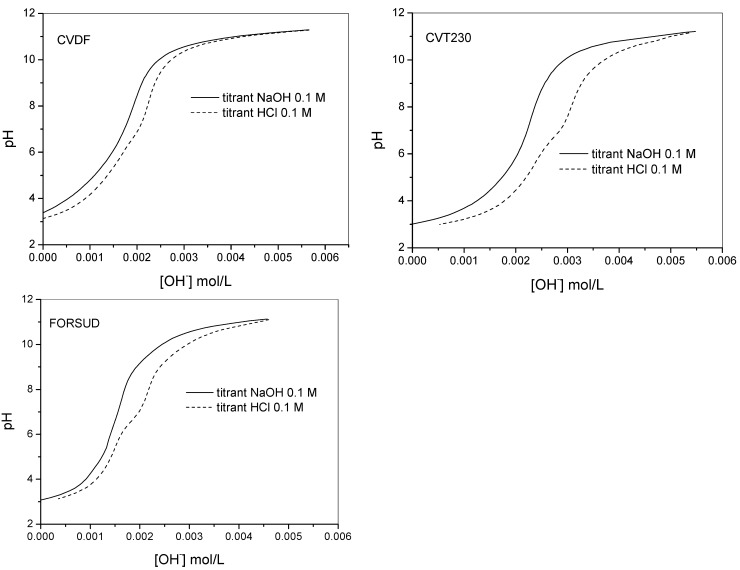
Direct (alkalimetric) and reverse (acidimetric) titration curves of CVDF, CVT230 and FORSUD at 0.5 g·L^−1^ concentration in 0.1 mol·L^−1^ TEACl.

The proton binding capacity of the SBOs were characterized first by alkalimetric/acidimetric titrations performed on sample solutions containing the products at 0.30–1.0 g·L^−1^ concentration. The potentiometric titrations were performed in the 2.8–11.5 pH range with 0.1 mol·L^−1^ aqueous KOH or HCl. At acid pH the SBOs precipitate. However, the attainment of the equilibrium conditions was not slowed down. Figure 1 shows an example of the typical alkalimetric titration curves recorded for each type of SBO at 0.5 g·L^−1^ concentration. The equilibrium conditions were difficult to reach in the pH range 5.5–8. Figure 2 reports the direct alkalimetric and reverse acidimetric titration curves for each SBO type. It is evident the hysteresis phenomenon. In alkalimetric titration only one inflection point is evident, while the reverse titration presents two inflection points at pH 8.0–8.5 and 5.0–5.5. Moreover, the back titration points are more acidic than the ones of the direct titration. This phenomenon is not yet entirely understood. Similar behavior was reported for HS [17,20,21]. A plausible explanation is the change of the macromolecule conformation during the titration. On the basis of the solution behavior of SBOs described in the previous paragraph, one can suppose that, in acidic conditions, as at the start of the direct titration, the single molecules were in the compact pseudomicellar coil conformation, while, during titration, the molecules acquired the expanded coil form. In this form, the acidic functional groups became more accessible by the titrant. This change in the acidic groups’ arrangement during the titration could be responsible of the slow equilibration. In the reverse titration, the rearrangement of the molecular conformation during the titrant addition could be different compared to that during the direct titration. This might explain the observed hysteresis.

#### 2.2.1. Protonation Constants

The alkalimetric titration results were elaborated in order to calculate the protonation constants and the concentration of protogenic groups. A diprotic-like model (model I and model II) [22] and a discrete model (model III), by Masini* et al.* [15] were applied. The models were compared for their capacity to fit the experimental titration curves, and thus to allow calculation of protonation constants, and evaluation of acidic sites concentration. The results are reported in Table 2. In the diprotic-like model application (model I), the protonation constants of a diprotic site were calculated elaborating the titration data in the pH range 2.8–6.5. A good value of the weighted standard deviation of the fit was obtained for all SBOs. The protonation constant of a less acidic site was also calculated widening the pH range at 10.5. In this case the result was satisfactory only for the FORSUD SBO. Possible reasons might be the presence of more than three acid-base active sites or a high effect of the dissociation degree on the protonation constant. To obtain a better fit of titration curves a widening of the model was applied, increasing the number of protogenic sites (model II). By comparison, model III supposes that a macromolecule contains a series of discrete sites with different independent dissociation constants. The results in Table 2 show that all models fit the experimental data. However, based on the values of the weighted standard deviation, model III is proven the best. This result was expected, since model III contains no concentration constraints. Figure 1 shows the comparison between the experimental titration points and the curves calculated with model III.

**Table 1:** Chemical data and critical micellar concentration (CMC) for soluble bio-organic substances (SBOs).

SBO	Ash (*w*/*w* %)	C (*w*/*w* %) ^a^	N (*w*/*w* %) ^a^	Aliphatic ^b^	NR ^b,c^	OR ^b,c^	Ar ^b^	PhOY ^b,d^	COY ^b,e^	CMC (g·L^−1^)
CVDF	0.56	48.5	5.2	0.31	0.06	0.16	0.23	0.12	0.11	2.76
CVT230	0.81	52.5	5.2	0.28	0.07	0.02	0.22	0.11	0.11	4.2
FORSUD	0.01	57.5	8.8	0.43	0.09	0.15	0.15	0.06	0.13	1.02

^a^ Concentrations values referred to dry matter; ^b^ C types and functional groups concentration as mole fraction of total organic C; ^c^ R = H, alkyl or phenyl; ^d^ Y = OH and/or OR; and ^e^ Y = OH and/or NR.

**Table 2:** Chemical models and protonation constants of acidic sites obtained from the elaboration of titration data.

SBO	Chemical Model	Acidic Site	logβ ^a^	log*K* ^a^	Site Concentration (mmol·g^−1^)
CVDF	Model I	Hcvdf1 ^b^	5.69 ± 0.02 ^c^		3.0 ± 0.4
		H_2_cvdf1	9.83 ± 0.03	4.14	
		Weighted standard deviation of the fit ^d^	2.14		
	Model II	Hcvdf1	5.42 ± 0.02 ^c^		2.6 ± 0.2
		H_2_cvdf1	9.41 ± 0.04	3.99	
		Hcvdf2	7.78 ± 0.04		0.8 ± 0.2
		Hcvdf3	10.23 ± 0.02		0.9 ± 0.1
		Weighted standard deviation of the fit	2.55		
	Model III	Hcvdf1	4.34 ± 0.02		1.70 ± 0.08
		Hcvdf2	6.03 ± 0.03		1.0 ± 0.1
		Hcvdf3	8.33 ± 0.04		0.7 ± 0.1
		Hcvdf4	10.40 ± 0.02		0.9 ± 0.2
		Weighted standard deviation of the fit	1.73		
CVT 230	Model I	Hcvt1	5.29 ± 0.01		3.0 ± 0.1
		H_2_cvt1	8.86 ± 0.04	3.57	
		Weighted standard deviation of the fit	2.43		
	Model II	Hcvt1	5.10 ± 0.02		2.70 ± 0.05
		H_2_cvt1	8.61 ± 0.06	3.51	
		Hcvt2	7.58 ± 0.04		0.8 ± 0.1
		Hcvt3	9.97 ± 0.03		1.3 ± 0.2
		Weighted standard deviation of the fit	3.03		
	Model III	Hcvt1	3.87 ± 0.01		1.76 ± 0.03
		Hcvt2	5.76 ± 0.02		1.07 ± 0.07
		Hcvt3	8.26 ± 0.04		0.71 ± 0.08
		Hcvt4	10.16 ± 0.02		1.2 ± 0.2
		Weighted standard deviation of the fit	1.75		
FORSUD	Model I	Hforsud1	6.12 ± 0.03		1.61 ± 0.02
		H_2_forsud1	10.32 ± 0.07	4.20	
		Weighted standard deviation of the fit	1.66		
	Model II	Hforsud1	6.37 ± 0.05		1.67 ± 0.04
		H_2_forsud1	10.65 ± 0.08	4.28	
		Hforsud2	9.34 ± 0.02		1.602 ± 0.004
		Weighted standard deviation of the fit	1.80		
	Model III	Hforsud1	4.50 ± 0.04		1.015 ± 0.006
		Hforsud2	6.77 ± 0.06		0.67 ± 0.04
		Hforsud3	9.37 ± 0.02		1.58 ± 0.01
		Weighted standard deviation of the fit	1.43		

^a^ The protonation constants are expressed as log of β_r_ = [AH_r_^−*n* + *r*^]/[A^−*n*^][H^+^]*^r^* or *K* = [AH*_r_*^−*n* + *r*^]/[H*_r_*_-1_A^−*n* + *r*−1^][H^+^]. The log*K* values are reported only when different from log*β*; ^b^ Different numbers refer to different acidic sites. The species can be monoprotic or diprotic. In the latter case the species completely protonated are indicated as H_2_A and the monoprotonated as HA; ^c^ ±standard deviation; and ^d^ Weight for each experimental point is given as *w* = 1/*s*^2^ [25].

#### 2.2.2. Protogenic Sites Concentrations

Table 2 reports the concentration of the protogenic groups obtained by the alkalimetric titration data elaborated applying the three models. It may be observed that the results obtained by the different models are in good accordance with each other in all cases.

The literature dealing with protonation of HS assumes that the total concentrations of COOH and PhOH functional groups can be obtained as the sum of the concentration values for the species with log*K*_a_ < 8 for COOH and with log*K*_a_ > 8 for PhOH [17]. In this case, the attribution of log*K* values to specific functional groups is not allowed because the biopolymers present amines or other nitrogen functions. These functions can contribute to the acid/base reactivity and have log*K* values dispersed in a wide range as a function of their aromatic or aliphatic nature. However, it is reasonable to suppose that the log*K*_a_ < 7 can be attribute to –COOH groups. Table 3 reports the concentration of –COOH calculated as the sum of the concentrations of the species with a log*K*_a_ < 7 by the application of Model III (Table 2).

To compare NMR and titration data, it should be known that the ^13^C NMR spectra of SBOs are characterized by rather broad bands [24]. These do not allow determining selectively concentrations of acidic groups but only total aromatic C bonded to OH or alkoxy or aryloxy moieties (PhOY) from the ^13^C signal at 140–160 ppm and total carboxyl C (COY) as sum of COOH and CON C from the ^13^C signal at 160–185 ppm. The contribution of ester C to the carboxyl C NMR signal was excluded, as these functional groups were expected to be hydrolyzed in the alkaline treatment of the SBO sourcing material. For these reasons, we expect that the concentration values of COY and PhOY obtained by ^13^C NMR are expected higher than those obtained by titration. This expectation was verified for both –COOH and PhOH, where PhOH is the sum of the concentration of all the species with a log*K*_a_ > 7, obtained by the application of Model III (Table 2), as usually done for HS.

The availability of the data in Table 2, coupled to the data in Table 1, allows obtaining the breakdown of the COY data in Table 1 into their components. Table 3 shows the results. These were obtained according to the assumption underlying the following Equations:

COY = COOH + CONR
(1)

N = NR + CONR
(2)

In these equations, COY and N are the total carboxyl and organic N concentration values in Table 1, with R = H, alkyl or aryl, while COOH is the concentration value obtained from titrations and calculated as reported above. The results obtained clearly show the different nature of FORSUD SBO compared to those of CVDF and CVT230 SBO. The former contains lower number and lower concentration of protogenic species than the other two SBOs. These features are likely to be related to the different sourcing materials. The CVDF and CVT230 SBOs are sourced from composted wastes. The higher number and concentration of protogenic species might be the result of the bio-oxidation conditions under which the sourcing CVDF and CVT230 materials were obtained. Moreover, it has been found out that FORSUD SBO has a higher content of amides (Table 3). The higher amount of these functional groups in FORSUD can be traced back to the different nature of its sourcing material relative to CVDF and CVT230. Indeed, coming from the organic humid fraction of urban wastes, fermented under anaerobic conditions, FORSUD is expected to be richer in proteinaceous matter originating from food wastes.

**Table 3:** Breakdown of NMR data into single functional groups concentrations (mmol·g^−1^).

SBO	PhOY ^a^	COY ^a^	CONR ^b^	NR ^b^	COOH ^c^
CVDF	4.84	4.55	1.85	1.85	2.7
CVT230	4.76	4.75	1.92	1.78	2.83
FORSUD	2.76	6.16	4.48	1.82	1.68

^a^ Data obtained by applying Equation (3) to values reported in Table 1. The acronyms are explained in the text, Section 2.2.2.; ^b^ Data calculated through Equations (1) and (2); ^c^ The concentrations of carboxylic groups were obtained from application of chemical modeling processes (model III) and the value reported is the sum of the concentrations of the species with a log*K*_a_ < 7 reported in Table 2.

## 3. Experimental Section

### 3.1. Chemicals

Tetraethyl ammonium chloride (TEACl, purity ≥ 98.5%), the concentrated acids HCl (37%) and HF (≥40%) were from Sigma Aldrich (St. Louis, MO, USA). Standard KOH and HCl solutions were prepared by diluting Merck (Darmstadt, Germania) or Sigma Aldrich concentrated products and standardized against potassium hydrogen phthalate (Sigma Aldrich, purity ≥ 99.5%) and sodium carbonate (Sigma Aldrich, purity ≥ 99.5%), respectively. Grade A glassware and deionized water were used for all the solutions.

### 3.2. Preparation and Characterization of SBOs

The SBOs were sourced from materials sampled from three different streams of the ACEA Pinerolese waste treatment plant in Pinerolo (Italy). These were the digestate (FORSUD) recovered from the plant biogas production reactor fed with the organic humid fraction from separate source collection of urban refuse, the compost (CV) obtained from urban private gardens and public park trimming residues aged for 230 days, and the compost (CVDF) obtained from a 35/55/10 *w*/*w*/*w* FORSUD/home gardening and parks trimming residues/sewage sludge mix aged for 110 days, composting under aerobic conditions. The sampled materials were processed in a pilot plant made available from Studio Chiono & Associati s.r.l. in Rivarolo Canavese, Italy. This comprised an electrically heated mechanically stirred 500 L reactor, a 102 cm long × 10.1 cm diameter polysulfone ultrafiltration membrane with 5 kDa molecular weight cut-off supplied by Idea Engineering s.r.l. from Lessona (Bi), Italy, and a forced ventilation drying oven. According to the operating experimental conditions, the materials sampled from the Acea plant were reacted 4 h with pH 13 water at 60 °C and 4 *v*/*w* water/solid ratio. The liquid/solid mix was allowed to settle to separate the supernatant liquid phase containing the soluble hydrolysate from the insoluble residue. The recovered hydrolysate was circulated at 40 L·h^−1^ flow rate through 5 kDa cut off polysulphone ultrafiltration membrane operating with tangential flow at 7 bar inlet and 4.5 bar outlet pressure to yield a retentate with 5%–10% dry matter content. The concentrated retentate was finally dried at 60 °C to yield the solid products in 15%–30% *w*/*w* yield, relatively to the sourcing material dry matter. These products were found to contain about 15%–28% ashes. The ashes contained, along with the added alkali cation of the hydrolyzing base, also other mineral elements such as Si, Ca, Mg, Al and Fe, which were present in the sourcing materials. To reduce mineral content, the solid SBOs were further washed with HCl, water, HF, again with water, and then dried at 60° to yield the final products which were used in this work. These are hereinafter named after their sourcing materials,* i.e.*, FORSUD, CVDF and CVT230. They were characterized according to a previously reported procedure [23] for the content of ash, elemental C and N, organic C types and functional groups, and critical micellar concentration in water solution. To compare the amount of acid groups obtained from ^13^C NMR spectra with those obtained by titrations, the maximum concentration (mmol·g^−1^) of carboxyl-like structures and phenolic groups is calculated by the Equation (3):

(3) $$C_{i}=\;\frac{C_{i}\;\times \;\%C_{EA}}{1.20011}$$

where *C_i_* is the mole fraction of carboxyl-like structures (δ = 160–190 ppm) or phenolic groups (δ = 140–160 ppm) from NMR analysis in the sample, and %*C*_EA_ is the weight percentage of carbon in the sample as determined by elemental analysis.

### 3.3. Electromotive Force Measurements

Potentiometric measurements were performed using a Metrohm mod. 713 potentiometer (resolution of ±0.1 mV) coupled with a Metrohm 665 Dosimat burette (minimum volume deliverable of ±0.001 cm^3^) and equipped with a Metrohm combined glass electrode (mod. 6.0222.100).

For all the potentiometric measurements the electrode couple was standardized, in terms of pH = −log[H^+^], by titrating HCl 10 mmol·L^−1^ solution (at the same ionic strength value as the solution under study) with standard KOH in order to determine the standard potential *E*^0^ before each experiment. All the potentiometric titrations were carried out in a stream of purified nitrogen gently bubbled in the titration cell to avoid O_2_ and CO_2_ contamination. The measurement cells were thermostated at 25 ± 0.1 °C by means of a water circulation from a thermocryostat (mod. D1-G Haake, Victoria, Australia). The experiments were carried out in TEACl aqueous solutions with ionic strength 0.1 mol·L^−1^. The TEACl was chosen as background salt because it does not interact significantly with polyelectrolytes [22]. Each titration was repeated at least twice.

In order to evaluate the acidic properties of SBOs, a series of alkalimetric titrations in 0.1 mol·L^−1^ TEACl aqueous solutions, was performed. The concentration of SBO in the solution to be titrated were from 0.30 to 1.0 g·L^−1^, lower than their critical micelle concentrations (Table 1) in order to better assess the number and the nature of SBO protogenic sites (see Section 2.1.). As the SBOs are soluble in high alkaline conditions, the sample was dissolved at 100 g·L^−1^ concentration in 1 mol·L^−1^ KOH. The total alkali content in the solution was in excess relative to the total acidic functions assuming that the maximum content of acid groups could not be greater than that reported in Table 1 for total carboxyl (COY, Y = OH and/or NR) and phenol (PhOY, Y = H and/or OR) groups estimated by ^13^C NMR spectroscopy. The direct titration of the acidic groups was performed on the 100 g·L^−1^ SBO solution after the dilution and the addition of 0.1 mol·L^−1^ HCl to pH~3.0. The acidified solution was then titrated with the 0.1 mol·L^−1^ KOH solution up to pH 11.5, in order to evaluate the protonation constants and the concentration of both carboxylic and phenolic functions. The reverse titrations were performed by addition of 0.1 mol·L^−1^ HCl. A total of 50 to 120 points was taken for each titration and the potential was read only when its variation was smaller than 0.5 mV·min^−1^. Under this condition, equilibrium was assumed to be reached before each successive alkali/acid addition. The total titration time was about 3 h.

### 3.4. Data Analysis and Calculations

To determine the electrode parameters from calibration data the calculations were performed by using the non-linear least squares computer program ESAB2M [26]. This program allowed to refine the analytical concentration of the reagents, the electrode formal potential *E*^0^, the coefficient *j_a_* relative to the acidic junction potential (*E_j_* = *j_a_* [H^+^]) and the ionic product of water *K*_w_.

The estimation of the protonation constants and sites concentrations was performed by the BSTAC [25] software elaborating the titration data recorded on SBO solutions. It employs an iterative and convergent numerical method, which is based upon the linear combination of the mass balance equations, minimizes the error squares sum on electromotive force values and takes into account eventual variations of ionic strength among and/or during titrations. The protonation constants are expressed by Equation (4),

*β*_r_ = [AH*_r_*^−*n* + *r*^]/[A^−*n*^][H^+^]*^r^* (4)

This equation is related to the equilibrium:

A^−*n*^+ *r*H^+^ ↔ AH*_r_*^−*n* + *r*^ (5)

where AH*_r_*^−*n* + *r*^ is a weak *r*-protic acid and A^−*n*^ is the corresponding deprotonated form.

The operator according to a trial and error procedure then selects the most probable species involved in the equilibria. The software refines the constants and the concentration of the acidic sites. Several chemical models were taken into consideration to determine the one fitting best the experimental data.

## 4. Conclusions

The elaboration of potentiometric data has allowed attaining consistent chemical models to assess the number, nature and concentration of acidic functions in SBO biopolymers sourced from the digestate and composts of urban biowastes. Three models have been used and all three fitted significantly the experimental data. Several protogenic sites with different acid strength have been found. This is consistent with the fact that SBOs, as products of biological origin, are mixtures of molecules with different chemical composition, molecular weight, structure and solution behavior. The observed hysteresis between tandem forward and reverse titration curves and the values of protonation constants calculated for protogenic functions confirm the similarity of the SBOs with natural humic substances. The alkalimetric titration data have been found consistent with the concentration values for total carboxyl and amine functional groups determined by ^13^C NMR spectroscopy. The titration data have allowed obtaining the breakdown of total carboxyl and N containing functional groups. Thus, a more detailed characterization of the above biomacromolecules has been possible.

The experimental approach taken in this work has been applied for the first time to the above SBO biopolymers derived from municipal biowaste treatment materials. It has been confirmed a useful tool to understand the nature of complex organic matter from biological source. For the specific case of SBOs, the herewith-reported results may turn useful to foresee and/or explain the behavior of these biomacromolecules in specific applications. The availability of acid-base sites with different strength is highly important in relation to the SBOs capacity to bind and keep metals at neutral and alkaline pH, and precipitate at acid pH. The metal binding capacity and solubility properties can be exploited for uses in different fields, such as agriculture and environmental remediation.
